# Aspirin for primary prevention of stroke in individuals without cardiovascular disease—A meta-analysis

**DOI:** 10.1177/1747493019858780

**Published:** 2019-06-25

**Authors:** Conor Judge, Sarah Ruttledge, Robert Murphy, Elaine Loughlin, Sarah Gorey, Maria Costello, Aoife Nolan, John Ferguson, Martin O Halloran, Michelle O'Canavan, Martin J O'Donnell

**Affiliations:** 1 HRB—Clinical Research Facility, NUI Galway, Galway, Ireland; 2 Translational Medical Device Lab, NUI Galway, Galway, Ireland; 3 Wellcome Trust—HRB, Irish Clinical Academic Training, NUI Galway, Galway, Ireland

**Keywords:** Stroke, aspirin, cardiovascular, prevention

## Abstract

**Background:**

The benefits of aspirin for primary prevention of stroke are uncertain.

**Methods:**

We performed a cumulative meta-analysis of trials investigating aspirin for primary prevention of cardiovascular disease with a focus on stroke. We assessed the effects of aspirin on non-fatal stroke, hemorrhagic stroke, non-fatal myocardial infarction, all-cause mortality, cardiovascular mortality, major gastrointestinal bleeding, and an analysis of net clinical effect, in populations without a history of clinical or subclinical cardiovascular disease.

**Summary of review results:**

Among 11 trials (157,054 participants), aspirin was not associated with a statistically significant reduction in non-fatal stroke (odds ratio, 0.94; 95% CI, 0.85 to 1.04) but was associated with an increased risk of hemorrhagic stroke (odds ratio, 1.29; 95% CI, 1.06 to 1.56). Aspirin was not associated with a statistically significant reduction in all-cause mortality (odds ratio, 0.97; 95% CI, 0.92 to 1.03) or cardiovascular mortality (odds ratio, 0.94; 95% CI, 0.85 to 1.03). Aspirin was associated with a reduction in non-fatal myocardial infarction (odds ratio, 0.80; 95% CI, 0.69 to 0.94) and an increased risk of major gastrointestinal bleeding (odds ratio, 1.83; 95% CI, 1.43 to 2.35). Using equal weighting for non-fatal events and major bleeding, we observed no net clinical benefit with aspirin use for primary prevention.

**Conclusion:**

Our meta-analysis reports no benefit of aspirin for primary stroke prevention.

## Introduction

Aspirin has been shown to be effective for secondary prevention post myocardial infarction and stroke,^1^ but there is uncertainty about its role in primary prevention populations, including those with cardiovascular risk factors (e.g. diabetes mellitus).^2^ While a large number of the general population elect to take a daily aspirin for primary prevention of stroke,^3^ there is disagreement in current guidelines about the use of aspirin for primary prevention.^4^ The guidelines are based on interpretation of previous meta-analytic findings, which report a modest benefit for selected high risk patients, mostly related to a small absolute reduction in non-fatal myocardial infarctions in elderly patients, which is offset by an increased risk of major gastrointestinal and hemorrhagic stroke.^5^ The American Heart Association and American Stroke Association's guidelines on primary prevention of stroke give a moderate IIa recommendation for aspirin's use in primary prevention of stroke in high risk groups.^6^ A survey of U.S. adults between 45 and 75 years showed that, of the people taking aspirin, 81% were taking aspirin for primary prevention of cardiovascular disease. Two-thirds of these aspirin users reported stroke prevention as the primary indication.^3^

Since the publication of the United States Preventive Services Task Force meta-analysis and recommendations for aspirin in primary prevention,^4^ additional large randomized control trials focusing on older adults,^7,8^ diabetes,^9^ and moderate cardiovascular risk^10^ have reported their results. In this meta-analysis of randomized controlled trials evaluating aspirin for primary prevention of cardiovascular disease, we sought to determine the summary effect of aspirin on primary prevention of stroke and other cardiovascular outcomes.

## Methods

### Cumulative meta-analysis

To reduce research waste,^11^ we (CJ and SR) extracted data from two previous meta-analyses: one of randomized controlled trials of aspirin in primary prevention of cardiovascular disease^12^ and the other of bleeding risks with aspirin for primary prevention of cardiovascular disease.^5^ We considered these meta-analyses of sufficiently high quality to avoid the need to repeat them. We limited our search to dates not included in these reviews (2015–2018). We (CJ and RM) repeated primary data extraction independently for all papers to confirm accuracy and resolved any inconsistencies by consensus (CJ, RM and SR).

### Selection criteria

We performed a systematic review according to published guidelines from the Cochrane Collaboration^13^ and Preferred Reporting Items for Systematic Reviews and Meta-Analyses (PRISMA).^14^ We selected randomized controlled trials of aspirin for primary prevention of cardiovascular disease. We included all trials with: participants older than eighteen years, evaluated aspirin therapy versus placebo, randomized controlled trials, blinded outcome assessment, no history of cardiovascular disease, greater than one-year follow-up and published as full reports. We did not exclude trials based on neuroimaging requirements for outcome assessment (stroke). We limited our search to published, peer-reviewed studies in English. The search was not limited to a patient group or aspirin dose.

### Search strategy

We developed a search strategy for the PUBMED and EMBASE databases (Supplementary Figures I and II). The databases were searched from January 2015 to November 2018. Two reviewers (CJ and RM) independently screened titles and abstracts using the Rayann web application.^15^ Full texts were sourced for relevant articles. Inclusion criteria were assessed independently, and the final list was agreed by consensus. We also screened the reference list of similar review articles and earlier published meta-analyses obtained in our search. The protocol for the systematic review was registered on PROSPERO, the international prospective register of systematic reviews.

### Data extraction

We used a standardized data collection form (available on request). For each study, we extracted the title, year of publication, aspirin dose, active and control numbers, non-fatal stroke and hemorrhagic stroke. We did not pre-specify a definition for stroke. Instead, we used the definition reported by each individual paper. We included both ischemic and hemorrhagic stroke in our definition of non-fatal stroke. We also extracted non-fatal myocardial infarction, all-cause mortality, cardiovascular mortality and major gastrointestinal bleeding. Reviewers (CJ, RM and SR) independently extracted data, compared for inconsistencies, and merged into a final data set.

### Data synthesis and analysis

We present a descriptive analysis of each individual trial (Table 1). We calculated odds ratio (OR) and 95% confidence intervals from individual studies. Weighted pooled treatment effects were calculated using a random effects model. The variability across studies due to heterogeneity was estimated with the I^2^ statistic. We calculated the incident density rate for each study outcome by dividing the event totals by the person years of follow-up. We meta-analyzed the incident density rates to obtain pooled estimates and their 95% confidence intervals. Net-benefit was calculated as the risk difference between the benefits of all-cause mortality and non-fatal events (myocardial infarction and stroke), minus the harm of increasing major gastrointestinal bleeding and hemorrhagic stroke. Statistical analysis was performed using the Metafor package^16^ on R Statistical Software (V3.4.3).

**Table 1.** Study description, stroke and other cardiovascular events.

Study Name	Dose (mg/day)	Followup (years)	Total Subjects	Mean Age (years)	Female (%)	DM (%)	Non-fatal Stroke (Aspirin)	Non-fatal Stroke (Control)	Haem. Stroke (Aspirin)	Haem. Stroke (Control)	All-cause Mortality (Aspirin)	All-cause Mortality (Control)	CV Mortality (Aspirin)	CV Mortality (Control)	Non-fatal Myocardial Infarction (Aspirin)	Non-fatal Myocardial Infarction (Control)	Major GI Bleeding (Aspirin)	Major GI Bleeding (Control)	Definition of GI Bleed
BMD, 27	500	6	5139	NR	0	2	61	27	13	6	270	151	119	59	80	41	3	3	Fatal GI bleeds
PHS I, 26	162.5	5	22071	NR	0	–	110	92	23	12	217	227	81	83	129	213	49	28	Required major transfusion, death
HOT, 21	75	3.8	18790	61.5	47	8	NR	NR	14	15	284	305	133	140	68	113	77	37	Major and Fatal
TPT, 22	75	6.8	2540	57.5	0	NR	16	25	2	0	113	110	49	49	47	73	6	2	Required transfusion and/or surgery
PPP, 23	100	3.6	4495	64.4	57.46	16.5	15	18	2	3	62	78	17	31	15	22	17	5	GI bleeding
WHS, 20	50	10.1	39876	54.6	100	2.6	198	244	51	41	609	642	120	126	184	181	129	94	Required transfusion or caused death
JPAD, 24	100	4.37	2539	64.5	45.37	100	27	27	5	3	34	38	1	10	12	9	4	0	Required a transfusion
JPPP, 25	100	5	14464	70.5	57.7	34	–	–	28	15	297	303	58	57	20	38	103	31	Serious bleeds requiring transfusion or hospitalisaiton
ARRIVE, 10	100	5	12546	63.9	29.55	0	–	–	8	11	160	161	38	39	88	98	4	2	
ASCEND, 9	100	7.4	15480	63.2	37.4	100	202	229	55	45	748	792	197	217	191	195	137	101	Transfusion or hospitalisation to control bleeding
ASPREE, 7	100	4.7	19114	74	56	10.8	195	203	42	32	558	494	78	81	156	168	162	102	Bleeding that led to transfusion, hospitalization, surgery, or death

## Results

In total, 11 randomized controlled trials were eligible that recruited 157,054 participants and reported 1920 non-fatal strokes and 426 hemorrhagic strokes. Additionally, there were 2141 non-fatal myocardial infarctions, 6653 deaths, 1783 cardiovascular deaths and 1096 major gastrointestinal bleeds. Our updated search results found 1841 studies from PUBMED and 1807 studies from EMBASE, 613 duplicate studies were removed, 3032 studies were excluded after title and abstract screening, leaving 3 studies for inclusion (Supplementary Figure III). Three of the trials included in previous meta-analysis were excluded due to prior cardiovascular disease, two trials with participants having peripheral vascular disease^17,18^ and one trial with nearly half having previous cardiovascular disease.^19^ Of the included trials, nine were trials of aspirin at a dose of 100 mg or less.^7,9,10,20–25^ The mean follow-up across all studies was 5.58 years. The mean age was 63.79 years. 39.13% of the participants were female.

### Non-fatal stroke

**Figure 1.**
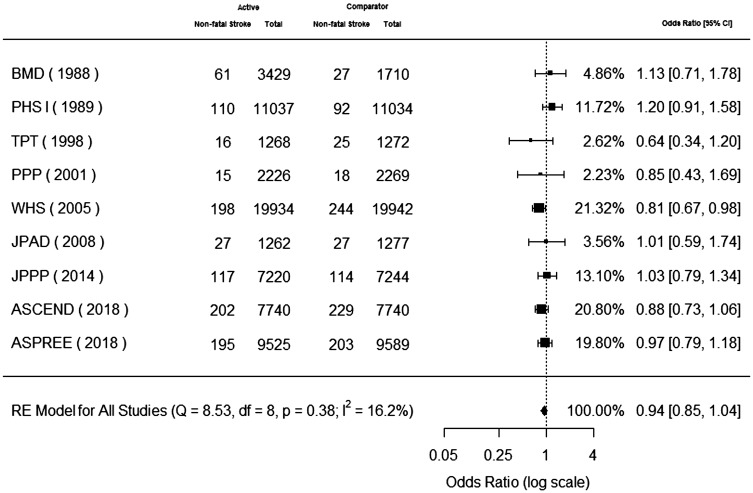
Aspirin for primary cardiovascular prevention and benefit for non-fatal stroke. Forest plot for non-fatal stroke. Forest plot showing the effect of aspirin therapy on non-fatal stroke. The squares and bars represent the mean values and 95% confidence intervals of the effect sizes, while the size of the squares reflects the weight of the studies. The combined effects appear as diamonds and the vertical dashed line represents the line of no effect.

Nine trials reported non-fatal stroke.^7,9,19,20,22–27^ Non-fatal stroke occurred in 941 (1.19%) patients in the aspirin group and 979 (1.26%) patients in the control group. Aspirin use for primary cardiovascular prevention was not associated with a significant decrease in non-fatal stroke (odds ratio, 0.94; 95% CI, 0.85 to 1.04) (Figure 1). The P value for heterogeneity was 0.38, I^2^ = 16.2%, Q = 8.53, and degrees of freedom = 8. A sensitivity analysis including only studies with imaging requirement for diagnosis of stroke^7,20,22,23,25^ was also non-significant for aspirin benefit on non-fatal stroke in primary prevention (odds ratio, 0.90; 95% CI, 0.79 to 1.02).

### Hemorrhagic stroke

**Figure 2.**
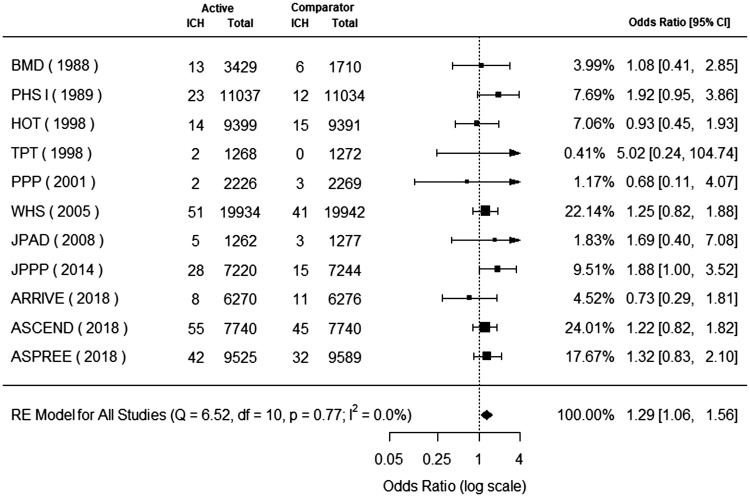
Aspirin for primary cardiovascular prevention and benefit for hemorrhagic stroke. Forest plot for hemorrhagic stroke. Forest plot showing the effect of aspirin therapy on hemorrhagic stroke. The squares and bars represent the mean values and 95% confidence intervals of the effect sizes, while the size of the squares reflects the weight of the studies. The combined effects appear as diamonds and the vertical dashed line represents the line of no effect.

Eleven trials reported hemorrhagic stroke.^7,9,10,20–27^ Hemorrhagic stroke occurred in 243 (0.30%) patients in the aspirin group and 183 (0.24%) patients in the control group. Aspirin use for primary cardiovascular prevention was associated with a significant increase in hemorrhagic stroke (odds ratio, 1.29; 95% CI, 1.06 to 1.56) (Figure 2). The P value for heterogeneity was 0.77, I^2^ = 0.0%, Q = 6.52, and degrees of freedom = 10.

### All stroke

Eleven trials reported all stroke.^7,9,10,20–27^ All stroke occurred in 1277 (1.61%) patients in the aspirin group and 1297 (1.67%) patients in the control group. Aspirin use for primary cardiovascular prevention was not associated with a significant decrease in all stroke (odds ratio, 0.95; 95% CI, 0.88 to 1.03). The P value for heterogeneity was 0.47, I^2^ = 2.4%, Q = 9.67, and degrees of freedom = 10.

### Non-fatal myocardial infarction

**Figure 3.**
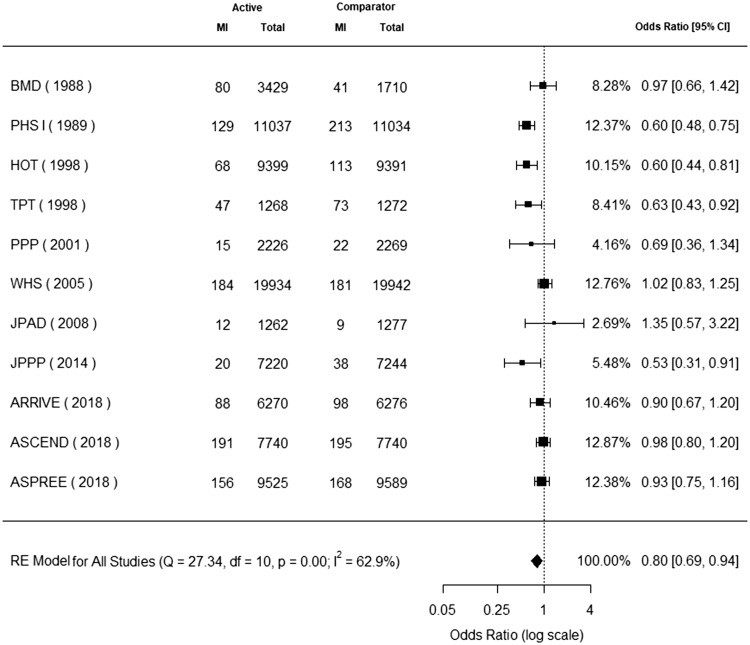
Aspirin for primary cardiovascular prevention and benefit for non-fatal myocardial infarction. Forest plot for non-fatal myocardial infarction. Forest plot showing the effect of aspirin therapy on non-fatal myocardial infarction. The squares and bars represent the mean values and 95% confidence intervals of the effect sizes, while the size of the squares reflects the weight of the studies. The combined effects appear as diamonds and the vertical dashed line represents the line of no effect.

Eleven trials reported non-fatal myocardial infarction.^7,9,10,19–27^ Non-fatal myocardial infarction occurred in 990 (1.25%) patients in the aspirin group and 1151 (1.48%) patients in the control group. Aspirin use for primary cardiovascular prevention was associated with a significant decrease in non-fatal myocardial infarction (odds ratio, 0.80; 95% CI, 0.69 to 0.94) (Figure 3). The P value for heterogeneity was 0.00, I^2^ = 62.9%, Q = 27.34, and degrees of freedom = 10.

### All-cause mortality

**Figure 4.**
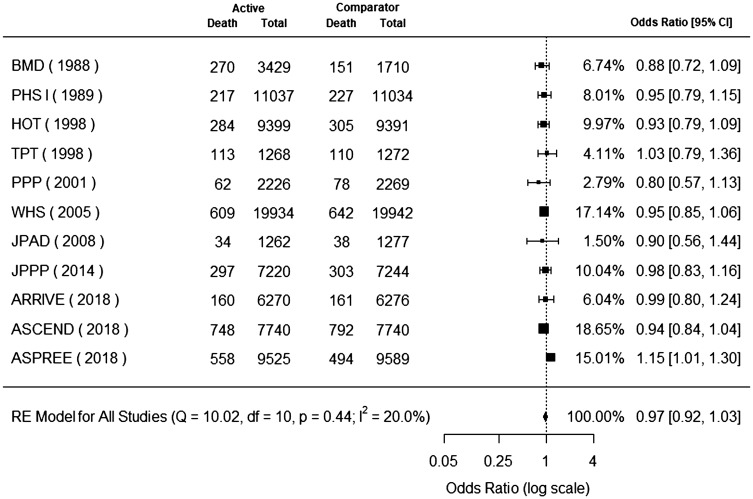
Aspirin for primary cardiovascular prevention and benefit for all-cause mortality. Forest plot for all-cause mortality. Forest plot showing the effect of aspirin therapy on all-cause mortality. The squares and bars represent the mean values and 95% confidence intervals of the effect sizes, while the size of the squares reflects the weight of the studies. The combined effects appear as diamonds and the vertical dashed line represents the line of no effect.

Eleven trials reported all-cause mortality.^7–10,19–27^ All-cause mortality occurred in 3352 (4.23%) patients in the aspirin group and 3301 (4.25%) patients in the control group. Aspirin use for primary cardiovascular prevention was not associated with a significant decrease in all-cause mortality (odds ratio, 0.97; 95% CI, 0.92 to 1.03) (Figure 4). The P value for heterogeneity was 0.44, I^2^ = 20.0%, Q = 10.02, and degrees of freedom = 10.

### Cardiovascular mortality

Eleven trials reported cardiovascular mortality.^7–10,17–27^ Cardiovascular mortality occurred in 891 (1.12%) patients in the aspirin group and 892 (1.15%) patients in the control group. Aspirin use for primary cardiovascular prevention was not associated with a significant decrease in cardiovascular mortality (odds ratio, 0.94; 95% CI, 0.85 to 1.03) (Supplementary Figure IV). The P value for heterogeneity was 0.60, I^2^ = 0.0%, Q = 8.29, and degrees of freedom = 10.

### Major gastrointestinal bleeding

Eleven trials reported major gastrointestinal bleeding.^7,9,10,20–27^ Major gastrointestinal bleeding occurred in 691 (0.87%) patients in the aspirin group and 405 (0.52%) patients in the control group. Aspirin use for primary cardiovascular prevention was associated with a significant increase in major gastrointestinal bleeding (odds ratio, 1.83; 95% CI, 1.43 to 2.35) (Supplementary Figure V). The P value for heterogeneity was 0.01, I^2^ = 61.5%, Q = 23.55, and degrees of freedom = 10.

### Net clinical effect

Table 2 reports the pooled estimates with confidence interval for population density incidence rates for non-fatal stroke and hemorrhagic stroke in the aspirin group, control group and the difference in incidence rates between the two groups. Non-fatal myocardial infarction, non-fatal stroke and major gastrointestinal bleeding are also reported. To determine a net clinical effect, we calculated the risk difference between benefit and harm, benefit from reduction in non-fatal stroke (0.16 per 1000 person years; 95% CI, −1.07 to 1.39) and non-fatal myocardial infarction (0.54 per 1000 person years; 95% CI, −0.83 to 1.91) and harm from increase in major gastrointestinal bleeding (−0.49 per 1000 person years; 95% CI, −1.23 to 0.25) and increase in hemorrhagic stroke (−0.12 per 1000 person years; 95% CI, −0.50 to 0.26). The overall net-benefit was non-significant at 0.09 per 1000 person years (95% CI, −1.93 to 2.11).

**Table 2.** Incidence rates per 1000 person years

	Aspirin		Control		Difference		
	Estimate	95% CI	Estimate	95% CI	Estimate	95% CI	NNT/NNH
Stroke							
Non-fatal stroke	2.59	1.84 to 3.63	2.75	2.01 to 3.77	0.16	−1.07 to 1.39	6250.00
Hemorrhagic stroke	0.51	0.36 to 0.72	0.39	0.28 to 0.54	−0.12	−0.50 to 0.26	−8333.33
Other outcomes							
Non-fatal myocardial infarction	2.24	1.53 to 3.29	2.78	1.89 to 4.08	0.54	−0.83 to 1.91	1851.85
Cardiovascular mortality	2.01	1.25 to 3.23	2.33	1.53 to 3.56	0.32	−1.05 to 1.69	3125.00
All-cause mortality	7.67	5.64 to 10.42	8.02	5.94 to 10.84	0.35	−3.02 to 3.72	2857.14
Major gastrointestinal bleeding	1.04	0.55 to 1.96	0.55	0.29 to 1.02	−0.49	−1.23 to 0.25	−2040.82

Note: The incidence rates for 1000 person years for non-fatal stroke, hemorrhagic stroke, non-fatal myocardial infarction, cardiovascular mortality, all-cause mortality and major gastrointestinal bleeding are reported. The incidence rates are reported for the aspirin group, control group and the difference between the two groups. The incidence rate was calculated by dividing the event totals by the person years of follow-up.

CI: confidence interval; NNT: number needed to treat; NNH: number needed to harm.

## Discussion

### Main findings

We performed a cumulative systematic review and meta-analysis of all randomized controlled trials of aspirin for primary prevention of cardiovascular disease to investigate the relationship between aspirin therapy and stroke. We did not find a statistically significant decreased risk for non-fatal stroke (odds ratio, 0.94, 95% CI, 0.85 to 1.04). We did find a significant increase in hemorrhagic stroke (odds ratio, 1.29; 95% CI, 1.06 to 1.56). There was no significant benefit for all-cause mortality (odds ratio, 0.97; 95% CI, 0.92 to 1.03) or cardiovascular mortality (odds ratio, 0.94; 95% CI, 0.85 to 1.03). We found a statistically significant decreased risk of non-fatal myocardial infarction (odds ratio, 0.80; 95% CI, 0.69 to 0.94) with aspirin use for primary prevention, but a commensurate increase in major gastrointestinal bleeding (odds ratio, 1.83; 95% CI, 1.43 to 2.35). Our net-benefit analysis showed no significant effect of aspirin on the composite of all-cause mortality, non-fatal stroke, non-fatal myocardial infarction and major bleeding.

Our updated review reports similar treatment estimates for non-fatal stroke and non-fatal myocardial infarction to previous meta-analysis of primary prevention populations (completed prior to recent published RCTs, ARRIVE, ASCEND and ASPREE)^12^ but differ for all-cause mortality. Previous meta-analyses have reported a small significant reduction in all-cause mortality but our updated results show no significant reduction in this outcome. One potential explanation for the reduction in pooled benefit of aspirin is that the earlier trials were performed in a time of suboptimal modifiable risk factor control especially blood pressure, smoking and hyperlipidemia. For example, the ASCEND and ASPREE trials had rates of statin use of 75% and 34% respectively and had low rates of current smoking, 8.3% and 4% respectively. A recent meta-analysis^28^ that included ARRIVE, ASCEND and ASPREE, reported significant reductions in ischemic stroke, with an increase in ICH. However, that meta-analysis included clinical trials of populations with subclinical cardiovascular disease i.e. participants with sub-clinical peripheral vascular disease.^17,18^ As such, our meta-analysis specifically addresses the net effect of aspirin on stroke outcomes in general populations without clinical or subclinical cardiovascular disease. Finally, similar to Zheng et al., we repeated the analysis for all stroke (fatal and non-fatal ischemic and hemorrhagic) but excluded participants with previous cardiovascular disease,^17,18^ the pooled estimate for reduction in all stroke remained non-significant (odds ratio, 0.95; 95% CI, 0.88 to 1.03) for aspirin use in primary prevention.

The current American Heart Association/American Stroke Association (AHA/ASA) guidelines give a class IIa recommendation to the use of aspirin for cardiovascular prevention (including but not specific to stroke) for patients at high risk (10-year risk > 10%). The results of this meta-analysis do not support this recommendation. There is now no overall net clinical benefit for aspirin in primary prevention.

### Strengths and limitations

There is substantial heterogeneity in population characteristics between the studies included including sex, age, and baseline co-morbidities. Two studies did not report stroke outcome and had to be excluded from the analysis, introducing a possible reporting bias. Three studies^21,26,27^ did not require imaging for diagnosis of stroke and three studies did not report if imaging was or was not used for diagnosis.^9,10,24^ This could introduce a misclassification bias and does not account for small strokes which may have been missed if MRI imaging was not used. A sensitivity analysis including only studies with imaging requirement for diagnosis of stroke^7,20,22,23,25^ remained non-significant for aspirin benefit on non-fatal stroke in primary prevention We also excluded three of the trials included in previous meta-analysis were excluded due to prior cardiovascular disease, two trials with participants having peripheral vascular disease^17,18^ and one trial with nearly half having previous cardiovascular disease.^19^ Excluding these trials gave a more precise answer to our research question, primary prevention of stroke with aspirin. This meta-analysis expands on previous work by including three recent large randomized control trials recently completed in varied populations, diabetic participants without history of cardiovascular disease, elderly patents (>70) and non-diabetic patients at moderate cardiovascular risk.

### Implications

In conclusion, there is no evidence of a reduction in non-fatal stroke for aspirin in primary prevention of cardiovascular events. There appears to be a small modest reduction in non-fatal myocardial infarction. Balancing this with an increased risk of major gastrointestinal bleeding and hemorrhagic stroke, there appears to be an even smaller net clinical benefit. Our findings do not support routine use of aspirin for primary prevention of cardiovascular events including stroke.

## Supplemental Material

Supplemental material for Aspirin for primary prevention of stroke in individuals without cardiovascular disease—A meta-analysis Click here for additional data file. Supplemental Material for Aspirin for primary prevention of stroke in individuals without cardiovascular disease—A meta-analysis by Conor Judge, Sarah Ruttledge, Robert Murphy, Elaine Loughlin, Sarah Gorey, Maria Costello, Aoife Nolan, John Ferguson, Martin O Halloran, Michelle O'Canavan and Martin J O'Donnell in International Journal of Stroke
