# Supplementary material for: Aspirin for primary prevention of stroke in individuals without cardiovascular disease—A meta-analysis
Source: Int J Stroke. 2019 Jun 25;15(1):9–17. doi: 10.1177/1747493019858780 (PMC7003154; doi:10.1177/1747493019858780)
Supplement: Supplemental material for Aspirin for primary prevention of stroke in individuals without cardiovascular disease—A meta-analysis [file Supplemental_Material.pdf]

## Supplementary Figure I – Pubmed Search Terms

Recent queries in pubmed

| Search | Query |
|---|---|
| #7 | Search ((#1) AND #2) AND #3 AND (("2015/01/01"[PDAT] : "2018/11/31"[PDAT]) AND "humans"[MeSH Terms]) |
| #6 | Search ((#1) AND #2) AND #3 Filters: Publication date from 2015/01/01 to 2018/11/31; Humans |
| #5 | Search ((#1) AND #2) AND #3 Filters: Publication date from 2015/01/01 to 2018/11/31 |
| #4 | Search ((#1) AND #2) AND #3 |
| #3 | Search prevent*[Title/Abstract] |
| #2 | Search ((aspirin[Title/Abstract]) OR acetylsalicylic acid[Title/Abstract]) OR Salicylate*[Title/Abstract] |
| #1 | Search (((((((cardiovascular disease*[Title/Abstract]) OR heart disease*[Title/Abstract]) OR myocardial infarction[Title/Abstract]) OR heart arrest[Title/Abstract]) OR myocardial isch*emia[Title/Abstract]) OR heart attack*[Title/Abstract]) OR stroke[Title/Abstract]) OR cerebrovascular disease[Title/Abstract]) OR cerebrovascular disorder*[Title/Abstract] |

# Supplementary Figure II – EMBASE Search Terms

| EMBASE Search | Query |
|---|---|
| #4 | Search (#1 AND #2 AND #3) AND [2015-2018]/py |
| #3 | Search 'prevent*':ab,ti |
| #2 | Search 'aspirin':ab,ti OR 'acetylsalicylic acid':ab,ti OR 'Salicylate*':ab,ti |
| #1 | Search 'cardiovascular disease*':ab,ti OR 'heart disease*':ab,ti OR 'myocardial infarction':ab,ti OR 'heart arrest':ab,ti OR 'myocardial isch*emia':ab,ti OR 'heart attack*':ab,ti OR 'stroke':ab,ti OR 'cerebrovascular disease':ab,ti OR 'cerebrovascular disorder*':ab,ti |

## Supplementary Figure III – PRISMA flow diagram

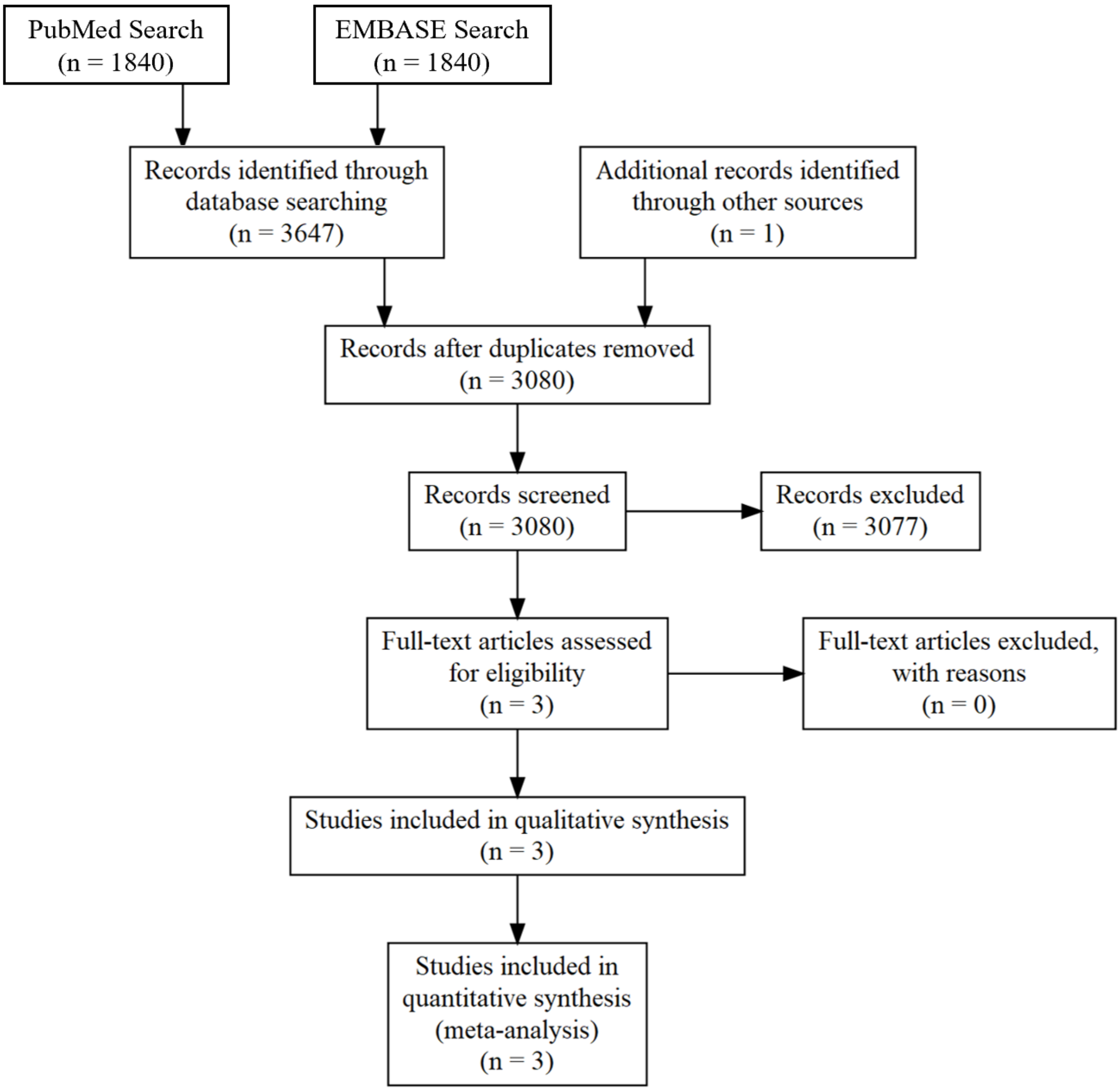


Supplementary Figure III – A PRISMA flow chart of our updated 2015-2018 search. Full-text articles were excluded due to not aspirin for primary prevention (n=3077).

Supplementary Figure IV– Aspirin for primary cardiovascular prevention and benefit for cardiovascular mortality

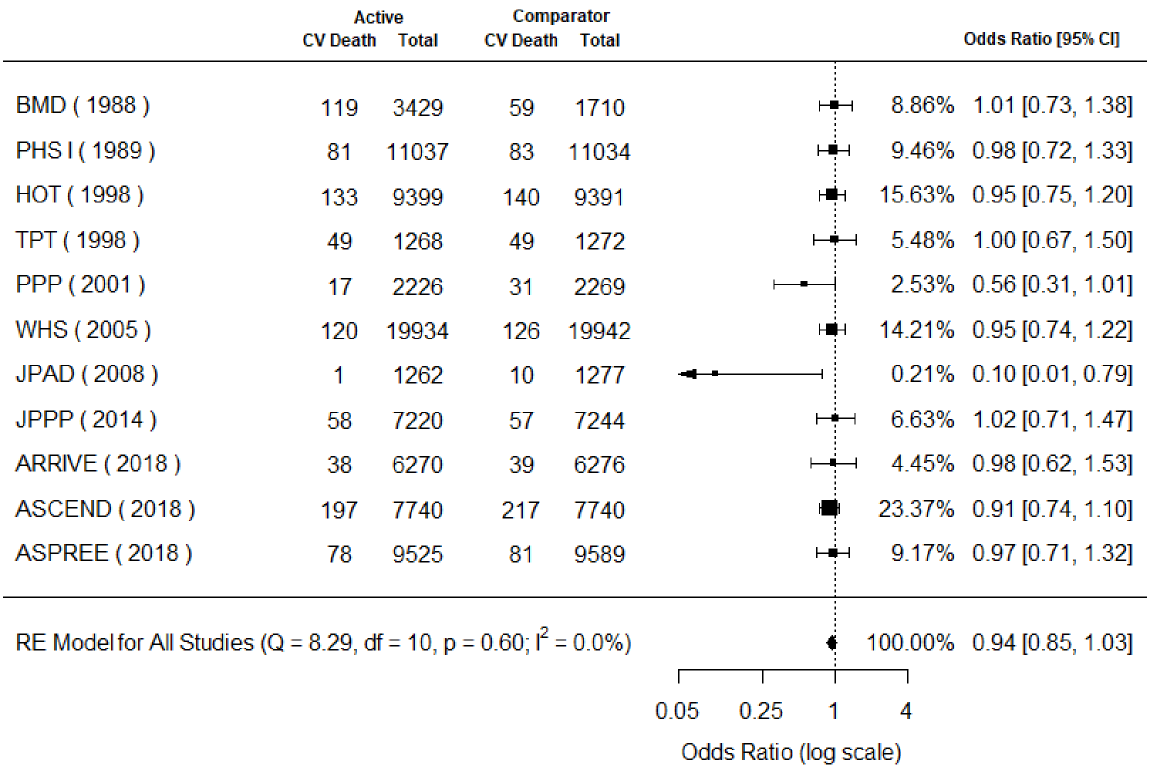


Supplementary Figure IV - Forest plot for cardiovascular mortality. Forest plot showing the effect of aspirin therapy on cardiovascular mortality. The squares and bars represent the mean values and 95% confidence intervals of the effect sizes, while the size of the squares reflects the weight of the studies. The combined effects appear as diamonds and the vertical dashed line represents the line of no effect.

## Supplementary Figure V – Aspirin for primary cardiovascular prevention and benefit for major gastrointestinal bleeding

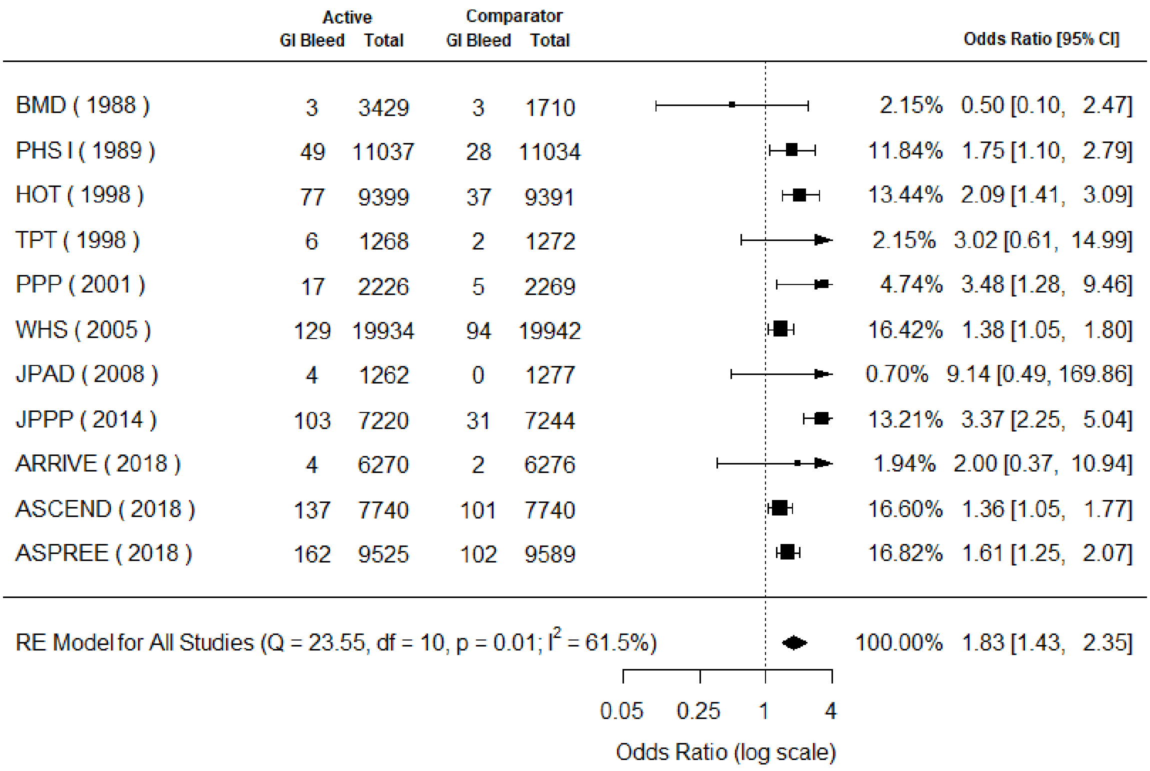


Supplementary Figure V - Forest plot for major gastrointestinal bleeding. Forest plot showing the effect of aspirin therapy on major gastrointestinal bleeding. The squares and bars represent the mean values and 95% confidence intervals of the effect sizes, while the size of the squares reflects the weight of the studies. The combined effects appear as diamonds and the vertical dashed line represents the line of no effect.
